# Advance decision-making in mental health – Suggestions for legal reform in England and Wales

**DOI:** 10.1016/j.ijlp.2019.02.002

**Published:** 2019

**Authors:** G.S. Owen, T. Gergel, L.A. Stephenson, O. Hussain, L. Rifkin, A. Ruck Keene

**Affiliations:** a Reader and lead, Mental Health, Ethics and Law Research Group, Institute of Psychiatry, Psychology and Neuroscience, King’s College London, 16 De Crespigny Park, London SE5 8AF; b Wellcome Senior Research Fellow, Mental Health, Ethics and Law Research Group, Institute of Psychiatry, Psychology and Neuroscience, King’s College London, 16 De Crespigny Park, London SE5 8AF; c Clinical Research Associate, Mental Health, Ethics and Law Research Group, ST6 General Adult Psychiatry and Medical Psychotherapy., Institute of Psychiatry, Psychology and Neuroscience, Institute of Psychiatry, Psychology and Neuroscience, King’s College London, 16 De Crespigny Park, London SE5 8AF; d ST4 in Forensic Psychiatry, South London and Maudsley NHS Foundation Trust, Denmark Hill, London SE5 8AZ; e Consultant Psychiatrist, Lambeth Home Treatment Team, South London and Maudsley NHS Foundation Trust, Denmark Hill, London SE5 8AZ, Visiting senior lecturer, Institute of Psychiatry, Psychology and Neuroscience, King’s College London 16 De Crespigny Park, London SE5 8AF; f Barrister, Wellcome Research Fellow, Visiting Lecturer at the Dickson Poon School of Law, Kings College London, Somerset House, East Wing WC2R 2LS, Visiting senior lecturer, Institute of Psychiatry, Psychology and Neuroscience, King’s College London 16 De Crespigny Park, London SE5 8AF

**Keywords:** Mental health act, Mental capacity act, CRPD, Advance decision making, Advance choice, Advance directive, Competence, Mental capacity

## Abstract

This paper argues that existing English and Welsh mental health legislation (The Mental Health Act 1983 (MHA)) should be changed to make provision for advance decision-making (ADM) within statute and makes detailed recommendations as to what should constitute this statutory provision. The recommendations seek to enable a culture change in relation to written statements made with capacity such that they are developed within mental health services and involve joint working on mental health requests as well as potential refusals. In formulating our recommendations, we consider the historical background of ADM, similarities and differences between physical and mental health, a taxonomy of ADM, the evidence base for mental health ADM, the ethics of ADM, the necessity for statutory ADM and the possibility of capacity based ‘fusion’ law on ADM. It is argued that the introduction of mental health ADM into the MHA will provide clarity within what has become a confusing area and will enable and promote the development and realisation of ADM as a form of self-determination. The paper originated as a report commissioned by, and submitted to, the UK Government's 2018 Independent Review of the Mental Health Act 1983.

## 1 Introduction

This paper argues that existing English and Welsh^1^ mental health legislation (the Mental Health Act 1983 (MHA)) should be changed to make provision for advance decision-making (ADM) within statute and makes recommendations as to what should constitute this statutory provision. It is hoped that ADM would be operationalised through the incorporation of formally recognised Advance Decision Making Documents (ADM-D) into statute. This will provide clarity within what has become a confusing area and will enable and promote the development and realisation of ADM as a form of self-determination. The paper originated as a report commissioned by, and submitted to, the UK Government's 2018 Independent Review of the Mental Health Act (IRMHA) (Wessely, Gilbert, Hedley, & Neuberger, 2018 p. 75, footnote 75). Recommendations contained in this paper have been used to formulate the concept of ‘advance choice documents’ and a ‘nominated person’ in the IRMHA report which government has pledged to bring forward in a new mental health bill (Department of Health and Social Care, 2018). The discussion and rationale behind these recommendations is therefore framed around English law. However, our analysis involves general concepts, makes international legal comparisons and draws on an international literature meaning that it has general relevance to the international debate on incorporation of ADM into mental health legislation.

There are high levels of agreement within health policy that ADM should be a goal and surveys show a generally positive attitude toward ADM amongst stakeholders (Bartlett, Mudigonda, Chopra, Morriss, & Jones, 2016; Elbogen et al., 2006; Gieselmann, Simon, Vollmann, & Schone-Seifert, 2018; Hindley et al., 2019; Swanson et al., 2003; Swanson, Swartz, Ferron, Elbogen, & Van Dorn, 2006). International (United Nations, 2006) and regional (Ward, 2017) human rights bodies strongly promote ADM on autonomy grounds. In England and Wales, the Mental Capacity Act (MCA), and, to a lesser extent, the Mental Health Act (MHA), already incorporate versions of ADM. Despite this, take up of ADM-D in mental health services is low.

Existing statutory provision for ADM in England within the MCA has been developed primarily in the context of physical healthcare, and, with limited exceptions, is not recognised within the MHA. We argue that, in order to meet ethical requirement for autonomy and to satisfy current service user demand, there should be some level of parity in terms of making provision for ADM-D within the MHA as well. However, we will also argue that parity of mental and physical healthcare is not the full story. Rather than straightforward fusion of MCA principles into the MHA, allowance must be made for some key differences between mental and physical healthcare and, while many aspects of the MCA's ADM provision can be adopted, some modifications are necessary.

In formulating our recommendations we considered the following topics: the historical background of ADM; similarities and differences between physical and mental health; a taxonomy of ADM; the evidence base for mental health ADM; the ethics of ADM; the necessity for statutory ADM and consideration of ‘fusion law’.

### 1.1 Aims

The recommendations made in this paper aim to:

i. Enable a culture change in relation to written statements made with capacity such that they are developed within mental health services and involve joint working on mental health requests as well as potential refusals.
ii. Enable mental health Advance Decisions to Refuse Treatment (ADRT) with limitations reflecting legitimate public interests.
iii. Develop mental health attorneys using a “nominated person” system
iv. Give service users more insurance that well thought through mental health ADM-Ds will be respected.

## 2 Historical background of ADM in mental and physical healthcare: parity and difference

Legal discussion of ADM has largely developed within a physical healthcare context which has reinforced the gap between mental and physical healthcare. From clinical, legal and ethical perspectives there are clear reasons to question this division and suggest there should be parity in making provision for ADM. At the same time, it is also important to recognise that certain differences remain between mental health ADM and physical health ADM which should be reflected in the specifics of ADM policy.

Historically, the sovereignty (or autonomy) principle in medical law was always related to the body. One of the most famous statements of the principle was made in 1914 by Justice Cardozo:

“Every human being of adult years and sound mind has a right to determine what shall be done with his own body” (Schloendorff v Society of New York Hospitals (1914) 211 NY 125)

This is a right to refuse rather than request medical treatment, based on what is recognised within medical ethics and law as the “fiduciary” nature of the doctor- patient relationship. The doctor offers the patient treatment that they, by the standards of professional practice, consider to be beneficial and the patient consents to it or refuses it. This right to refuse, but not to request, treatment has been repeated often in the English courts (e.g. Airedale NHS Trust v Bland (1993) AC 789) and is foundational to medical law.

Also to be noted here is the “soundness of mind” exclusion, which has been key to the development of ADM. Similar exclusions can be found in older ethical writings, such as Mill's 1869 *On Liberty*. Here Mill states that “over himself, over his own body and mind, the individual is sovereign”, but adds that this

is meant to apply only to human beings in the maturity of their faculties…Those who are still in a state to require being taken care of by others, must be protected against their own actions as well as against external injury (Mill, 1839).

Article 5 of the post-WWII European Convention on Human Rights (ECHR) states the unsound mind exclusion in these terms:

Everyone has the right to liberty and security of person. No one shall be deprived of his liberty save in the following cases and in accordance with a procedure prescribed by law:

…

e. the lawful detention of persons for the prevention of the spreading of infectious diseases, of persons of unsound mind, alcoholics or drug addicts or vagrants; (Council of Europe, 1950 p. 8).

As can be seen, the historical development of the autonomy principle in healthcare has tended to bifurcate the right to refuse treatment into the adult of sound mind and the adult of unsound mind, conceptualising the former as the ‘medical patient’, the latter as the ‘psychiatric patient’. For the latter, the right to refuse treatment is not recognised, although the unsound mind concept was criticised for implying a lasting or comprehensive state of incapacity. The Royal Commission on the Law Relating to Mental Illness and Mental Deficiency 1954–1957 chaired by Lord Percy concluded that:

the term “person of unsound mind” is also criticised, as it gives many people the false impression that it implies a state of permanent mental instability. It was never meant to carry any such implication, and many of the patients to whom it has to be applied…are expected to, and do, recover quickly from their illnesses (Percy Commission, 1957 Cmnd. 169, p. 59).

Percy's Royal Commission introduced the concept of “mental disorder” as alternative terminology for the psychiatric patient which remains to this day in the MHA.

The law has also developed in two different directions, reflecting this psychiatric/medical divide. For the ‘medical patient’, where right to refuse treatment was accepted, it also became increasingly necessary for medical law to specify what it meant by “unsoundness of mind” in order to determine which consents and refusals were owed legal respect (i.e. where the courts had no jurisdiction to interfere) and which were not. This stream of legal thinking developed the concept of mental capacity and the English courts started to address this for treatment in detail in the early 1990s starting with a case called Re. C ((Adult Refusal of Treatment) [1994] 1 WLR 290) (Ruck Keene, Kane, Kim, & Owen, 2019). Running together with these discussions about mental capacity, or decision-making capacity (DMC), was a discussion about advance decision-making. If a person had an absolute right to refuse medical treatment when they had mental capacity to decide it, why could that person not extend that right to a future time (e.g. when in a coma) when they lacked mental capacity to decide. The case law developed this notion as an ‘advance decision to refuse treatment’ (ADRT) and, by the early 2000s, both the concept of DMC and ADRT were embedded in the English case law. Other discussions extended an existing legal provision for advance decision making in property and affairs – the power to appoint an enduring power of attorney - to health and welfare. This became a lasting power of attorney (LPA) for health and welfare. The attorney, appointed by the patient when they had DMC to appoint, substituted for the patient when the patient lost DMC to decide treatment and had the right to consent to or refuse treatment as if the patient. Both ADRTs and LPAs for health and welfare could apply to life sustaining treatment and the procedures laid out were simple and largely administrative.

The Law Commission consolidated the case law in a series of recommendations which became the Mental Capacity Act 2005. The ADM concepts in the MCA are detailed in Section 4.

For the ‘psychiatric patient’, there was the MHA, laying down the criteria under which involuntary treatment for mental disorder would be sanctioned. The MHA responded to the MCA rules on ADM with amendments made in the MHA 2007. In general, it was made clear that ADM did not apply to compulsory treatment for mental disorder under the MHA (see Section 4 below). ADM was allowed, with limits, in relation to ECT and community treatment orders and, in its code of practice, it promoted ‘wishes expressed in advance’.

Outside the law, mental health clinical practice developed ADM stimulated by patient centred care movements. These were informal kinds of ADM such as crisis plans, joint crisis plans, advance care plans which are detailed in Section 4 below - typically communicating information about what to do in situations when planning was considered helpful such as mental health crises, perinatal or palliative care. The informal kind of ADM most studied in England is the ‘joint crisis plan’. This evolved out of the ‘crisis plan’ which had its origins in the mental health survivors' movement. The evidence base surrounding this is reviewed in Section 5.

### 2.1 Questioning the distinction between ‘medical’ and ‘psychiatric’ patient

Although the law has developed along the lines of a ‘medical’/’psychiatric’ division, it is helpful to question, from clinical, legal and ethical perspectives, whether such a distinction remains valid or helpful.

From the clinical perspective, patterns of health and healthcare are changing. Service users with severe psychiatric problems are increasingly recognised as having medical problems and shorter life expectancies due to medical conditions (Ilyas, Chesney, & Patel, 2017). There are also medical complications of psychiatric treatments, notably metabolic disease from antipsychotic medications. Being a ‘psychiatric patient’ can go alongside being a ‘medical patient’ in clinical contexts such as dementia and delirium, while psychiatric problems, such as depression, are common in chronic medical conditions or at end of life. It would be difficult to imagine, a contemporary GP, for example, conceptualising their patients as *either* medical *or* psychiatric patients with much success. For all these patients, both mental and physical health decision making are relevant. One patient is faced with an ADM task, whether for mental and/or physical health decisions – a situation reflected in the fact that informal care plans and crisis plans often cover both mental health and physical health matters.

From the legal perspective, it has become clear that the MCA can apply to ‘psychiatric’, as well as ‘medical’ patients and to mental, as well as physical, health decisions. The MCA has also clarified that legal compulsory powers (often associated with the ‘psychiatric patient’) extend to treatment of ‘medical patients’. Further breakdowns of the distinction include the legal fact that ‘psychiatric’ patients under the MHA are already considered in the same legal terms as ‘medical patients’ in relation to some mental health treatments such as ECT and that the courts are increasingly analysing ‘psychiatric patients’, in the same legal terms as ‘medical patients’, i.e. by reference to the question of whether they have or lack DMC and, if they lack DMC, what forms of medical treatment for mental disorder are in their best interests (e.g. A local authority v E and others [2012] EWHC 1639 (COP); Nottinghamshire Healthcare MHS Trust vs RC [2014] EWCOP 1317; An NHS Trust v Ms. X [2014] EWCOP 35). Similar patterns are occurring outside courts with the MCA now being routinely applied in self harm presentations in A&E contexts and increasingly in psychiatric inpatient contexts (e.g. regarding discharge).

From an ethical/philosophical perspective the distinction also looks problematic. It is not clear why the status of being ‘a psychiatric patient’ should a priori exclude the right to self-determination (the basic effect in Mill, Cardoza, article 5 ECHR). The bifurcation of the health and social care universe into physical health versus mental health treatments involves an outdated mind/body dualism well known to philosophers (and also to liaison psychiatrists working in medical hospitals). Therefore, it is more helpful to ask: what are the similarities and differences between ADM in mental health and physical health?

## 3 Mental health and physical health ADM: similarities and differences

Terms such as “ADM”, “mental health/illness”, “physical health/illness” have a variety of meanings, so we begin with some working definitions for clarification. Here, we use: ‘mental health ADM’ to refer to any decision made about one's mental health treatment (e.g. medical/psychological) and care for a future time when one is mentally ill; ‘physical health ADM’ to refer to any decision made about one's physical health treatment (e.g. medical/surgical) and care for a future time when one is physically ill.

We use “mental illness” to mean any health problem significantly affecting the mind regardless of cause (e.g. concepts and categories in books and journals of psychiatry, clinical psychology, official mental disorder taxonomies). By “physical illness” we mean any health problem significantly affecting the body regardless of cause (e.g. concepts and categories in books and journals of medicine and surgery, official taxonomies of cardiology, oncology, infectious diseases, respiratory medicine, etc.).^2^

With these working definitions in mind, we now turn to the similarities and differences between mental health and physical health ADM.

### 3.1 Similarities

We consider similarities to include the following:

3.1.1 *A concept of decision-making capacity (DMC)*. Both mental and physical health ADM typically require, whether implicitly or explicitly, a concept of DMC. In effect whether P or P's ADM decides (or helps to decide) depends upon whether P retains or is lacking in DMC. In practice, clear cut DMC can be hard to evidence – especially in retrospect.
3.1.2 *Forecasting*. Both require forecasting future illness and treatment scenarios and projecting oneself into those scenarios. This may be hard if one has no previous experience of the scenarios or treatments (e.g. complex treatments for an illness one has never had) or relatively easy (e.g. discrete treatments which one has had before for an illness one already lives with).
3.1.3 *A personal contemplation of loss of DMC*. Both require contemplating future periods of illness and future periods of incapacity. This may be challenging as contemplating future difficulties can be emotionally demanding and illicit normal avoidance.
3.1.4 *Knowledge of healthcare services*. Both require projecting oneself into healthcare services that exist (rather than services one may wish to exist). This requires contextual knowledge of healthcare possibilities and constraints.
3.1.5 *Self-determination*. Both are exercises in personal autonomy applied to one's own health.

### 3.2 Differences^3^

We have considered differences to include:

3.2.1 *Fluctuating DMC*. This is more significant for mental health ADM due to the cyclical nature of illness and concomitant capacity loss in conditions such as bipolar and psychosis. Repeated episodes of mental state change and treatment provide opportunities to learn what treatment may be wanted in future episodes. This learning may naturally lead to advance treatment and care requests.
3.2.2 *Anticipating marked changes in expressed will and preferences about treatment and care*. Again, this is more significant in mental health ADM than physical health ADM and is related to the phenomenon of loss of awareness of mental illness. Consider a person with bipolar who also has a cardiac problem. The person transitions into a manic episode. The loss of awareness is (typically) centred on being manic, rather than on having a cardiac problem. The effect is that the person, when well, has to anticipate themselves making more objection to mental health treatment than to physical health treatment during the period where DMC to decide treatment is lost. This means that the idea of “self-binding” over one's future objection is more relevant in mental health ADM than physical health ADM.
3.2.3 *Life sustaining ADRT*. It would be very difficult to get an ethical or policy consensus on enabling people to die of mental illness through advance decisions to refuse mental health treatment. The Wooltorton case is illustrative of this dilemma. Kerrie Wooltorton was a 26 year old woman known to mental health services with an emotionally unstable (or borderline) personality disorder and a history of self-harm with compulsory treatment under the MHA. She sought to take her life in 2007 by swallowing antifreeze, and although she had herself called an ambulance and had allowed herself to be taken to hospital, she refused the medical treatment which would have saved her life. Her written statement on arrival at hospital read:
  *To whom this may concern, if I come into hospital regarding taking an overdose or any attempt on my life, I would like for NO lifesaving treatment to be given. I would appreciate if you could continue to give medicines to help relieve my discomfort, painkillers, oxygen,* etc. *I would hope these wishes will be carried out without loads of questioning.*
  *Please be assured that I am 100% aware of the consequences of this and the probable outcome of drinking anti-freeze,* e.g. *death in 95–99% of cases and if I survive then kidney failure, I understand and accept them and will take 100% responsibility for this decision.*
  *I am aware that you may think that because I call the ambulance I therefore want treatment. THIS IS NOT THE CASE! I do however want to be comfortable as nobody want to die alone and scared and without going into details there are loads of reasons I do not want to die at home which I will realise that you will not understand and I apologise for this.*
  *Please understand that I definitely don't want any form of ventilation, resuscitation or dialysis, these are my wishes, please respect and carry them out.*
  She was considered to have capacity to refuse treatment under the MCA and died in hospital. The coroner made no criticisms of the actions of the medical staff and regarded them as consistent with the MCA although he did not consider the possibility that the medical treatment was for mental disorder and whether the MHA should have been used to treat without consent under S.63. Although on the facts this was not an ADRT case because she was assessed to have capacity to refuse on presentation, with small clinical and administrative variations, it could have been. The case generated intense debate and was raised as an early day motion in the UK House of Commons which criticised the Mental Capacity Act and called for amendments to prevent future cases (David et al., 2010; Dobbin, 2009).
3.2.4 *Third party harm ADRT*. Mental health ADRT resulting in third party harms is likely to raise different public interests than physical health ADRT because of the possibility of a direct relationship between mental illness and third-party harms. For example, if a person were to make ADRT for all antipsychotic treatment and then to become violent whilst psychotic this would raise distinctive public interests (Solomon, O'Reilly, Gray, & Nikolic, 2008). The Starson v. Swayze, [2003] 1 S.C.R. 722 [Starson] case is illustrative of this dilemma. The case involved Scott Jeffery Starson a man with paranoid schizophrenia who by 2005 had been continuously detained in Ontario psychiatric hospitals for nearly seven years without treatment on the basis that his death threats to others made it impossible for him to be discharged but his capacity to refuse treatment made him untreatable. Starson's psychiatrists believed that he was not capable and wanted to treat him with the standard antipsychotic medications. Legal clarification of his capacity to refuse took 5 years and in 2003, a majority of the Supreme Court of Canada upheld Starson's position that he had been capable to refuse the proposed treatment in 1998 and to make an advance refusal of antipsychotic treatment. Starson's mental health deteriorated particularly after 2003 developing paranoid delusions that if he ate or drank too much his imaginary son would be tortured. Starson's weight fell to 118 pounds and he became dehydrated to the point that he was at imminent risk of kidney failure and death. He was eventually treated on the basis that his capacity was likely to have changed since the Supreme Court ruling and because his mother, who was considered his proxy, wished for it. This individual case shows: a) the unintended consequences of separating the criteria for admission (risk to others) from treatment (incapacity and best interests), b) the controversy of allowing a very mentally ill person to die because of their refusal of treatment for mental illness (see also 3.2.3 above) and c) the importance of consistent statutory positions about harm to others due to mental illness as a ground for compulsory mental health inpatient treatment

## 4 A taxonomy of ADM

Before going on to review more broadly the case for mental health ADM and make further suggestions for reform, it is useful to review the current laws surrounding ADM within the UK and elsewhere.

### 4.1 Informal and statutory ADM

ADM-Ds belong to two kinds: informal and statutory. Informal kinds of ADM-Ds include care plans, crisis plans, joint crisis plans and self-binding plans, and their features are shown in Table 1. They are all plans for on-going management of mainly relapsing and remitting mental illnesses. None are legally binding or recognised specifically in any statute. Some, such as the care plan, have operated for a long time within clinical services and others, such as the crisis plan, have their origins outside clinical services (originally in ‘survivor movements’) which sought to build informal, non-clinical, support arrangements for times of crisis. The joint crisis plan, for which there is the largest empirical literature (see Section 5 below), was developed as a middle way between the crisis plan and the care plan. It involves an independent facilitator to mediate between service user and the clinician to generate the ADM-D. The concept of DMC is implicit or explicit, to varying degrees, in these informal ADM-Ds but there is a general idea that the ADM-D is made when a person with mental illness is comparatively well for application during periods when they are unwell.

**Table 1:** Informal ADMs.

	Purpose	Required parties involved (*preferable inclusions*)	Capacity assessment required (MCA)	Legally Binding for MH Services	Point at which effective	Requirements to alter/cancel plan
Care Plan	On-going management of a condition in the short/medium term	MH services Service user *(Family/Friends/Carers)*	No	No	On formulation of plan – ideally with agreement between all parties	Can be altered at any time – ideally with agreement between all parties
Joint Crisis Plan	Prospective management of a possible crises by identifying early interventions and treatment preferences	Independent facilitator MH services Service User *(Family/Friends) (Independent Advocates)*	No	No	At a time of crisis when service user retains ability to enact agreed plan	Can be altered at any time – ideally with agreement between all parties
Crisis Plan	Prospective management of a possible crises by identifying support available	MH services Service user *(Family/Friends) (Independent Advocates)*	No	No	At a time of crisis when service user retains ability to enact agreed plan	Can be altered at any time – ideally with agreement between all parties
Advance Care Plan	Prospective management of a chronic condition in the long term, including management of relapses. Made when service user is well.	MH services Service user *(Family/Friends) (Independent Advocates)*	No – although some models include a form of capacity assessment	No	At a time when the service user becomes unwell/ suffers a relapse	Can be altered at any time – ideally with agreement between all parties
Self-binding advanced care plan “Ulysses contracts”	Prospective management of a fluctuating condition focused on period of impairment/incapacity when service user anticipates their treatment preferences will change. Made when service user is well.	MH Services Service user *(Family/Friends) (Independent Advocates)*	No although some models stress capacity assessment.	No	At the time when the service user becomes unwell/losses capacity and preferences start to change	Can be altered/cancelled by service user at any time when well/with capacity. Altering treatment requests requires agreement with MH services.

Statutory kinds of ADM-D are also heterogeneous both within and across jurisdictions. This makes it important to define the specific ADM-D law is referring to and be clear on what it legally enables and does not enable. We present statutory ADM-D as they stand in England and Wales as at December 2018 and select some other jurisdictions for comparative purposes. Strictly, some of them do not have to be written documents (e.g. they can be verbal instructions) but for simplicity we will refer to all of them as ADM-Ds. We have selected Scotland and Northern Ireland because they have different ADM-Ds to England but are jurisdictions within the United Kingdom. We have also selected India because it is the jurisdiction showing the most recent innovations with mental health ADM-D and has aimed to achieve compliance with the Convention on the Rights of Persons with Disabilities.

### 4.2 England

Table 2 shows the features of statutory ADM-Ds in England. The MCA introduced 3 types of ADM-D: 1) written statements made with capacity, 2) advance decisions to refuse treatment (ADRT) (including life sustaining) and 3) Lasting Power of attorney for health and welfare (LPA) (including decision making authority over life sustaining treatment). All these ADM-Ds are based on DMC – in other words, they are made when a person has DMC to decide their health and welfare for periods when loss of capacity to do so is anticipated.

**Table 2:** Statutory ADMs in England and Wales.

England and Wales – Mental Capacity Act	Purpose	Required Parties Involved (*preferable inclusions*)	Capacity Assessment Required (MCA)	Legally Binding for MH Services	Point at which effective	Requirements to alter/cancel plan
Advance Decision to Refuse Treatment (ADRT) MCA (S.24–25)	Prospective management of a condition in the long term/future. Made when service user has capacity to make the decision (presumed). Only applies to refusals of treatment.	Service User Witness *(MH Services) (Family/Friends) (Independent Advocates)*	Service User is presumed to have capacity unless obvious reason to doubt it.	**Yes:** ADRT is binding on treatment (outside the scope of the MHA) if it (1) exists (i.e. made with capacity, and without duress/undue influence); (2) is valid; and (3) applicable to the treatment. If applies to ECT/other such forms of treatment (S58A MHA) and CTOs, regardless if detained under MHA. Can be overridden as per urgent treatment (S62 for ECT and S64G for CTOs) **No:** If detained under MHA and ADRT applies to MH care, (For ECT see above).	When a service user is judged not to have capacity to decide on relevant matters (MCA).	If no reason to doubt capacity then can be altered at any time via the same process as creating one. Can be challenged on grounds of existence/validity/applicability (trinity)
				Not binding in relation to Part 4 MHA treatment but Code of Practice and Case law have significant weight. Subsequent LPA covering same medical treatment will override any ADRT (see LPA section below)		
LPA – Health and Welfare MCA (S9-14)	Appointment of an individual/s as an attorney to decide on health and welfare matters when the service user lacks capacity to do so. Can Include ‘Preferences’, which Attorneys should bear in mind. Can include ‘Instructions’, which Attorneys MUST adhere to.	Donor Attorney/s Witness Certificate Provider (known to Donor for at least 2 years OR Individual with professional skills to judge understanding) Office of Public Guardian *(MH Services) (Replacement Attorney) (Family/Friends)* *(Independent Advocates)*	Donor is presumed to have capacity unless obvious reason to doubt it. Certificate Provider confirms that Donor understands significance of LPA	**Yes:** LPA is binding on treatment (outside the scope of the MHA) if it (1) exists (i.e. made with capacity, and without duress/undue influence); (2) is valid; and (3) applicable to the treatment. **No:** If decision related to medical treatment for mental disorder in MHA. Outside the MHA decision to refuse life-sustaining treatment unless expressly authorised by donor.	When a service user is judged not to have capacity (MCA).	If no reason to doubt capacity then can be cancelled by Donor through witnessed deed. Deed then sent to Office of Public Guardian. Can be challenged on grounds of existence/validity/applicability (trinity) Court can override attorney if felt that attorney is not acting in the best interests of donor
				Note: If valid and applicable ADRT exists in advance of LPA, attorney cannot override the ADRT unless LPA specifies attorney has the ability to do so. If LPA covers the same ground as an ADRT it automatically invalidates the ADRT.		
Advance Statement (written statement made with capacity) MCA (S.4(6)(a))	Prospective management of a condition in the long term/future made by a service user, usually when they are well Can relate to refusal of matter other than medical treatment (therefore not falling within ADRT). Can relate to request for treatment (or something else). Could be an ADRT which fails on technical grounds but is still a written statement.	Service User *(MH Services) (Family/Friends) (Independent Advocates)*	Service User is presumed to have capacity unless obvious reason to doubt it.	**No** However must be particularly considered (as far as reasonably ascertainable).	At any point after the statement has been made when the service user is judged not to have capacity (MCA).	Can be altered at any time when the person has capacity (presumed).

The MCA also introduces the concepts of “existence”, “validity” and “applicability” for ADRTs and LPAs. These concepts have specific legal meanings. “Existence” refers to an ADM-D being made by an adult (18 years and older) with DMC. “Validity” refers to the ADM-D being the most recent wish made by the adult with DMC. “Applicability” refers to the ADM-D applying to the current circumstances when DMC has been lost. The MCA provides that, for ADRT and LPA to be applicable to life sustaining treatment, it must be witnessed and contain a statement by the person to the effect that it is to apply to that treatment even if life is at risk,

The MHA only recognises ADRT and LPA in relation to electroconvulsive therapy (ECT) and community treatment orders (CTO). Otherwise the MHA makes no mention of ADM and Section 63 makes it clear that the consent of a patient shall not be required for any medical treatment for mental disorder if the treatment is given by or under the direction of the approved clinician. ADRT and has an “urgency” exclusion in relation to ECT and an “emergency” exclusion in relation to CTO - otherwise ADRT and LPA are legally binding.

Whilst not in the MHA itself, the most recent MHA code of practice (Department of Health, 2015) contains a chapter (chapter 9) on “wishes expressed in advance” which includes statements about DMC. The following are relevant extracts from the code of practice:

*1.8 Patients should be encouraged and supported to develop advance statements of wishes and feeling and express their views about future care and treatment when they are well.*
*9.1 Advance statements and decisions strengthen patients' participation in their treatment and recovery and help them to feel more empowered about what may happen to them should they lack mental capacity to make decisions about their care and treatment in the future.*
*9.4 Clinicians must consider advance statements when determining what is in the patient's best interests if the patient subsequently loses capacity.*
*9.17 Patients should be made aware that expressing their preference for a particular form of treatment or care in advance like this does not legally compel professionals to meet that preference. However, professionals should make all practicable efforts to comply with these preferences and explain to patients why their preferences have not been followed.*

So the legal landscape on ADM in England and Wales is currently complex. The MCA enables ADM in 3 different ways. The MHA disables ADM through part IV Section 63 though there are complexities in part IV in relation to ECT and CTO. The MHA does not recognise written statements made with capacity, but its Code of Practice encourages consideration of wishes expressed in advance with capacity.

### 4.3 Scotland

Scotland, unlike England and Wales, has no ADM in its Adults with Incapacity (Scotland) Act 2000, but has developed ADM in its Mental Health (Care and Treatment) (Scotland) Act 2003, which was amended in 2015. So, in Scotland ADM only exists, in statute, for mental health. It does not exist in statute for physical health where, instead, the common law is relied upon.

The Mental Health (Care and Treatment) (Scotland) Act 2003 has two kinds of mental health ADM-D: 1) advance statements and 2) named persons. Table 3 shows their features. Both require DMC assessments. Neither are legally binding, but a system of certification requires that the Mental Health and Welfare Commission hold a registry of all advance statements. If a clinical decision maker overrides an advance statement, they need to give reasons in writing to the patient and to the Commission. However, if a clinical decision maker overrides the views of a named person there is no duty to give reasons to the commission and we understand there is no central registry of named persons.

**Table 3:** Statutory ADMs in Scotland.

Scotland – Mental Health (Care and Treatment Act) (2003)	Purpose	Required Parties Involved (*preferable inclusions*)	Capacity Assessment Required	Legally Binding for MH Services	Point at which effective	Requirements to alter/cancel plan
Advance Statement (Scotland)	Prospective management of a condition in the long term/future made by a service user when they have the capacity to do so. Mental Health and Welfare Commission hold register of all advance statements.	Service User Witness (‘within class of persons’ – Psychologist/Medical Practitioner/OT/Nurse/Social Worker/Solicitor/Person employed in provision of care service) *(MH Services) (Named Person) (Family and Friends) (Independent Advocates)*	**Yes** – by Witness (Prescribed person)	**No:** Medical practitioner must show ‘*regard*’ to past or present wishes and feelings, may be expressed in form of advance statement or other. Overriding the advance statement either by medical practitioner or tribunal requires recording the circumstances and the rational as well as supplying the record to person who made statement, named person, welfare attorney, person's guardian, Commission. Mental health and Welfare Commission monitors promotion and uptake of advance statements.	At any point after the statement has been made including when the service user is judged not to have capacity and in relation to compulsory treatment.	Requires same process of making an advance statement, including a capacity assessment.
Named Person (Scotland)	Appointment of an individual as a Named Person to represent and safeguard the interests of an individual should they be subject to compulsory powers. Named Person does NOT replace service user in any way. Named Person has right to put forward own view, even if different to service user. Named Persons have right to documentation and speak at mental health tribunals. Note: Default Named Person abolished in 2015 reforms.	Nominator Named Person Witness (‘within class of persons’ – Psychologist/Medical Practitioner/OT/Nurse/Social Worker/Solicitor/Person employed in provision of care service) *(MH Services) (Family and Friends) (Independent Advocates)*	**Yes** – by Witness (Prescribed person)	**No:** Mental Health Officer, when practical, ascertain the name/address of Named Person before deciding whether to consent to granting of a short-term detention certificate.	When Named Person appointed and nominator is subject to compulsory powers.	Alteration/revoking requires same process of nominating a Named Person, including a capacity assessment. Medical Practitioner/Service User/Welfare Attorney/Guardian/relative/other person having an interest in welfare of service user can apply to Tribunal. Tribunal can appoint a Named Person instead of acting Named Person

### 4.4 Northern Ireland

Northern Ireland has enacted, but not yet brought substantially into force, a “fused” mental health law covering mental health and physical health ADM in one single statute: The Mental Capacity Act (Northern Ireland) 2016. This is modelled on the MCA. Table 4 shows the features. As well as the 3 MCA types of ADM-D (see Section 4.2) it also includes a nominated person, which is similar to the Scottish ‘named person’. LPAs (including life sustaining) and ADRTs (including life sustaining) are legally binding if existing, valid and applicable and no distinction is made between mental health and physical health ADM. Nominated persons, however, are not legally binding. The act also includes a requirement that the law for ADRT be reviewed by 2019. This review is underway at the time of writing.

**Table 4:** Statutory ADMs in Northern Ireland.

Northern Ireland – Mental Capacity Act 2016	Purpose	Required Parties Involved (*preferable inclusions*)	Capacity Assessment Required (MCA)	Legally Binding for MH Services	Point at which effective	Requirements to alter/cancel plan
Advance Decision (ADRT).	Prospective management of a condition in the long term/future. Made when service user has capacity to make the decision (presumed). Only applies to refusals of treatment.	Service User Witness *(MH Services)* *(Family/Friends)* *(Independent Advocates)*	Service User is presumed to have capacity unless reason to doubt it.	**Yes:** If deemed incapacitous under MCA and deemed valid and applicable. Including mental health matters. **No:** If subsequent LPA covering same ground as ADRT, has the effect of deeming donor to have withdrawn ADRT	When a service user is judged not to have capacity (MCA).	If no valid reason to doubt capacity then can be altered. Can be challenged on grounds of existence/validity/applicability via common law not in statute.
LPA – Health and Welfare	Appointment of an individual/s as an attorney to decide on health and welfare matters when the service user lacks capacity to do so. Can Include ‘Preferences’, which Attorneys should bear in mind. Can include ‘Instructions’, which Attorneys MUST adhere to.	Donor Attorney/s Witness Certificate Provider (Person of a Prescribed Description) Office of Public Guardian *(MH Services) (Replacement Attorney) (Family/Friends) (Independent Advocates)*	Donor is presumed to have capacity unless reason to doubt it. Certificate Provider confirms that Donor understands significance of LPA	**Yes:** If deemed incapacitous under MCA. Including mental health matters. **No:** Decision of giving/refusing life-sustaining treatment unless expressly provided. Doesn't authorise an attorney to deprive the donor of their liberty. Decision in respects to psychosurgery. Note: If subsequent LPA covering same ground as ADRT, has the effect of deeming donor to have withdrawn ADRT	When a service user is judged not to have capacity (MCA).	If no valid reason to doubt capacity then can be cancelled by Donor through witnessed deed. Deed then sent to Office of Public Guardian.
Nominated Person	Appointment of an individual (over the age of 16) to be involved in decision making in regards to prospective care. Note: If no individual is ‘nominated’ a default nominated person is appointed as per a pre-defined list in the Act. Service user can declare for an individual from list not be nominated person	Service User Nominated Person Witness (Prescribed Description) *(MH Services) (Family/Friends)* *(Independent Advocates)*	Witness of Prescribed Description certifies that individual understands the effect and not under undue pressure.	**No:** A duty to consult (if practicable/appropriate) and take into account views in determining best interests. A duty to inform in regards to individual.	After the appointment of the Nominated Person. Including when individual no longer has capacity.	Alteration/revoking requires same process of appointing a Nominated Person, including a capacity assessment. Tribunal can appoint/revoke a nominated person

Note: NI do not have statutory provisions about existence/validity/applicability but have reference to common law relating to ADRTs.

Northern Ireland – Mental Capacity Act 2016.

Review of law relating to advance decisions.

284.—(1) Before the third anniversary of the day this section comes into operation, the Department must—.

(a) review the law relating to advance decisions to refuse treatment; and

(b) produce a report setting out the conclusions reached on the review (including any proposals for changes to that law).

(2) The Department must lay a copy of the report before the Assembly.

### 4.5 India

India only has ADM in its Mental Health Care Act 2017, although physical healthcare ADM is available under common law (Ruck Keene, 2018). So, like Scotland, India has mental health ADM, but not physical health ADM, on its statute books. The Mental Health Care Act 2017 sought to achieve compliance with the CRPD and introduced a right to a mental health advance directive. Table 5 shows the features of mental health advance directive. It is based on DMC and includes both the right to request and to refuse treatment. Advance directives are binding except in emergencies or following successful applications to a board who must use specific criteria to judge the matter. The right to a mental health advance directive is a judiciable right.

**Table 5:** Statutory ADMs in India.

Mental Health Care Act 2017 - India	Purpose	Required parties involved (*preferable inclusions*)	Capacity assessment required	Legally binding for MH services	Point at which effective	Requirements to alter/cancel plan
Advance Directives	Prospective management of a condition in the long term/future. Made when service user has capacity to make the decision. Applies to requests and refusals of treatment for a mental illness.	Follows similar process for making a Will.	**Yes:** requires medical attestation that capacity is present at time of creating advance directive.	**Yes:** but does not apply to *‘emergency treatment – prevent death or irreversible harm, inflicting serious harm to himself or others, damage to property belonging to himself or to others where such behavior is believed to flow directly from the person's mental illness. Emergency treatment includes transportation of person with mental illness to a nearest mental health establishment for assessment’*	When a service user is judged not to have capacity	Requires the same process of creating an advance directive. Ideally with agreement between all parties. In addition to above ‘Board’ may review/alter/modify/cancel advance directives but only based on specific grounds. Board decision can be appealed to High Court of the State.
		Person (not a minor) 2 Witnesses Medical Practitioner (Capacity Attestation) Central Authority/Board *(MH Services) (Nominated Representative) (Family/Friends)*				

## 5 Making the case for mental health ADM: empirical evidence

The issue of what constitutes evidence for mental health ADM is not straightforward, given that it is not clear whether ADM is a rational or empirical concept and what constitutes a desirable outcome for ADM. If ADM is an inherent autonomy good, one might see uptake of ADM as the right outcome measure. If ADM is about self-determination, might the outcome measure be the availability of a supported and functioning ADM mechanism for all those who choose to take it up? Or service user satisfaction? If we see ADM as a mental health intervention, might demonstration of reduced compulsory treatment be the right outcome?

Moreover, given that ADM in the MCA was not evidence-based before it became accepted policy, could it be argued that the need for a specific mental health ADM evidence-base is redundant because it is a self-standing right? Is there a discrimination question potentially lurking here insofar as the evidence standard for mental health ADM may be set higher than the standard for physical health ADM?

We will examine the existing empirical literature, in terms of three broad questions, to allow for different perspectives on what counts as evidence.

### 5.1 What is the evidence people with mental illness want mental health ADM?

Although representative surveys are difficult to achieve in hard to reach groups, all the surveys that have been conducted on the views of those with SMI on ADM suggest that the majority of respondents are in favour.

Most data is from USA with sampling from community mental health services for severe mental illness (Swanson et al., 2006; Swanson, Swartz, Ferron, et al. (2006); Swanson et al., 2003; Van Dorn, Swanson, & Swartz, 2009). In all of these studies a majority (>50%) express positive views about mental health ADM. In England a Mental Health Alliance survey of 1218 people with mental illness who had experience of MHA detention found that 889 (73%) thought ADRT should be the same under MHA as MCA (Mental Health Alliance, 2017). A survey of 932 subscribers to Bipolar UK with experience of Bipolar (Hindley et al., 2019) found that 88% wanted any ADM and that 69% wanted self-binding ADM with collaboration with a psychiatrist. Studies report a large mismatch between actuality (what people with mental illness are doing) and aspiration (what they would like to do) on ADM. One survey of 544 people with Bipolar in England and Wales found 74.1% believed advance planning to be important but only 4–11% used any of the available legal provision to do so (Morriss, Mudigonda, Bartlett, Chopra, & Jones, 2017). A similar picture is emerging across Europe (Gieselmann et al., 2018).

### 5.2 What is the evidence mental health ADM-Ds are clinically feasible?

#### 5.2.1 Is the content of mental health ADMs clinically feasible?

Srebnik et al. (2005) studied the content of 106 mental health ADM-Ds in Washington State, USA. These were completed by community outpatients with experience of psychiatric hospitalisation and contained a mix of requested and refused medication. Two thirds refused ECT, while nearly all recognised a need for hospital in some circumstances. 95% of the ADM-Ds were rated as clinically feasible by independent psychiatrists.

Swanson, Swartz, Ferron, et al. (2006); Swanson, Swartz, Elbogen, et al., 2006 studied the content of 136 mental health ADM-Ds in North Carolina USA. This was a sample of people with severe mental illness in public community treatment programmes. No ADM-D refused all treatment. Most refused some medications and expressed a preference on which hospital for inpatient treatment. Independent psychiatrists judged 91% clinically feasible in relation to medication and 83% feasible in relation to hospitalisation. 94% of the ADM-Ds were regarded as having clinically useful information.

Farrelly et al. (2014) conducted a thematic analysis of 221 joint crisis plans that were drafted as part of the CRIMSON study (Section 5.3.2). These involved people with severe mental illness who were in community mental health teams in England. The majority of plans requested home treatment and treatment with respect and compassion from familiar clinicians. Around a half refused treatments, the majority of which were specific medications. Reilly and Atkinson (2010) comment that, while a common clinical concern is that patients will refuse all treatment, ‘research in both the USA and England suggests that it is very uncommon for people to refuse all medication’ (Reilly & Atkinson, 2010 p. 116).

Hindley et al. (2019) studied subscribers to Bipolar UK. Of 337 respondents with Bipolar who had mental health ADM-Ds, 40% requested specific meds, 25% refused specific meds, 31% refused ECT (5% requested), 35% requested hospitalisation and 68% specified a surrogate. Psychiatrists were involved in creating only 14% of these ADM-Ds (whereas 70% of services users wanted psychiatrist involvement in creating an ADM-D).

#### 5.2.2 Can ADMs be facilitated within clinical services?

In research contexts, uptake of mental health ADM-D is achieved in around 30–50% of those eligible.

Swanson, Swartz, Elbogen, et al. (2006); Swanson, Swartz, Ferron, et al. (2006) found, using a randomised control trial, that trained research assistants increased ADM-D uptake in people with severe mental illness compared to written information. Easter, Swanson, Robertson, Moser, and Swartz (2017), using a random allocation method, found evidence that facilitation by peers can achieve uptake similar to clinicians in people with severe mental illness. In the CRIMSON trial (Section 5.3.2) clinician engagement was found to be variable. Farrelly et al. (2014) explored the difficulties in securing clinical engagement with joint crisis plans. These included ambivalence about care planning, a perception that their clinical practice already used shared decision making tools and concerns about whether service user choices would be appropriate or realistic.

### 5.3 What is the evidence mental health ADM-Ds achieve positive clinical outcomes?

#### 5.3.1 Systematic reviews of Randomised Controlled Trials (RCTs)

Most of the RCT evidence addresses joint crisis plans and has measured reduction in compulsory treatment as the primary outcome. There is a problem of study heterogeneity and some variation between the conclusions of systematic reviews assessing the same studies. Campbell and Kiselly (2009) concludes there is too little data available to make definite recommendations. De Jong et al. (2016) includes two additional larger studies, which individually report no overall reduction in compulsory treatment, and conclude, somewhat confusingly, that there is a clinically significant reduction in compulsory admissions. A systematic review and meta-analysis commissioned by the IRMHA reported that crisis planning interventions, such as advance directives, reduced compulsory admissions but not voluntary or overall admissions (Molyneaux et al., 2018).

#### 5.3.2 Individual studies

The most comparable studies are the Henderson et al. (Henderson et al., 2004) and the CRIMSON multi centre RCT of joint crisis plans (Thornicroft et al., 2013). These were both studies conducted in England involving patients in community mental health teams with severe mental illness. Both used a similar joint crisis plan intervention and reported evidence for improved working alliance. However, the positive result of the first, in terms of a reduction of compulsory treatment, was not repeated in the second. The key difference between the two studies appears to be clinical buy-in. This was greater in Henderson et al. (Henderson et al., 2004), where the joint crisis plan facilitator was a senior mental health nurse known to most of the mental health community teams within a catchment area and most clinicians demonstrated involvement in the shared decision making. In the CRIMSON study (Thornicroft et al., 2013), a large, multi-centre study where the facilitators were less senior and unknown to the community teams, there was evidence of significant buy-out from the clinicians. Although, in a classical evidence hierarchy, the second larger study would trump the first, an important difference in conditions surrounding the complex interventions within the studies was clinical buy-in. The evidence seems to suggest that clinical buy-in may be necessary for reducing compulsory treatment in joint crisis plans and that clinical buy-in is easier to achieve in smaller RCTs where relationships exist between researchers and clinical teams and facilitators and clinical teams.

## 6 Making the case for ADM: ethical arguments

The idea of ADM – either with the legal analysis of ADRT or with the service user and clinical interest in a more person-centred healthcare – has been connected to the ethical concept of personal autonomy. Personal autonomy has been a major element within moral philosophy and medical ethics post war and the literature in recent years has extended discussion to social, or relational, aspects of autonomy. But, at its core, the concept of personal autonomy is a view of human action; actions are viewed as autonomous if the decision-making process underlying them is rooted in self-determination. This has been much discussed as a key principle and central right within international covenants of medical law, medical ethics and the disability rights movements.

### 6.1 Autonomy and precedent autonomy

From the Kantian perspective, autonomy is a universal ethical duty. Even if there are differences between decision-making about mental health and physical health there should be ethical parity in terms of this duty of self-determination which, on a Kantian view, must be universalisable. The Kantian view of autonomy does not equate autonomy with individual choice – it has to be action bound by the categorical imperative: “act in such a way that you treat humanity, whether in your own person or in the person of another, always at the same time as an end and never simply as a means” (Kant, 2002). From the Millian perspective individual choice is what matters most but it is choice, as we have seen in Section 2 above, that must be made by adults in the maturity of their faculties which does not bring harm to others.

Contemporary ethics and modern medical ethics develop this idea that autonomy, though importantly about choice (or supported choice), is not only about choice. Ethicists acknowledge a distinction between choice made with decision-making abilities – abilities needed to make a decision for oneself (to make the decision one's own and to be accountable for it) – and choice made without these abilities. In this sense, modern ethical concepts of autonomy blend key aspects of Kant's and Mill's conceptions.

Individual autonomy also involves “precedent autonomy” – this is the idea that autonomy can be reasonably extended to periods when one loses autonomy in order to maximise it overall, or give full expression to it (Dworkin, 2011). Precedent autonomy is the ethical basis for ADM in general and it presupposes not only autonomy but also, its flip side, loss of autonomy. Precedent autonomy acknowledges that preferences expressed with DMC about what happens at a future time without DMC can have ethical priority over preferences expressed at this future time without DMC. Although in some mental illnesses such as dementia there has been ethical debate about whether personality can change to such a degree, and without reversal, that precedent autonomy loses much of its ethical force (Dresser, 1995). Often the classical image of Homer's Odyssey (or Ulysses) is used to convey the idea of precedent autonomy: Odysseus knew his decision-making powers would succumb to the powers of the bewitching sirens as he guided his boat past them, and he knew his preference to sail far from the treacherous rocks would change as he heard their voices. So he exercised precedent autonomy by binding himself to the mast and getting promises from his sailors to plug their ears with wax and refuse to unbind him until they had sailed safely by. The modern application of this idea is the management of manic episodes using a self-binding ADM-D – an idea whose ethical and legal basis has been analysed (Gergel & Owen, 2015) and which has support in a Bipolar UK survey of attitudes to ADM (see Section 4 above).

### 6.2 Risk: personal and public interests

The concept of harm features widely in ethics and in human rights. For example, J.S. Mill's harm principle and classic statements of the rights of man. Examples of the latter include France's Declaration of the Rights of Man and of the Citizen of 1789 which states: “Liberty consists in the freedom to do everything which injures no one else; hence the exercise of the natural rights of each man has no limits except those which assure to the other members of the society the enjoyment of the same rights. These limits can only be determined by law.” (National Assembly of France, 1789, Article 4). These positions focus on harm to others as freedom's limit. “Other” may mean individuals (as in Mill's principle) or the public body/public interest (as in Rousseau's' “general will” that inspired the famous human rights statements from the French Revolution). But medical ethics has also widely discussed situations where the person is a child or a vulnerable adult where unnecessary harm may be brought upon *themselves* without the protective intervention from others. This is the concept of harm to self or health.

These are all public interest perspectives on harm. But the concept of harm can also be approached from the personal perspective – indeed the idea that a person with experience of illness should have a voice in what counts as harmful for them follows naturally from general notions of self-determination. For example, in one qualitative study of retrospective patient views on the justification of their involuntary treatment, 25/28 (89%) of patients belonging to a group who had positive views cited averting risk and feeling safe in hospital as justifying reasons. In the group who had ambivalent views on justification 10/12 (80%) cited these reasons too (Katsakou et al., 2012).

The concept of risk (likelihood of harm) features centrally in the MHA where risk to self, health or others is a criterion for compulsory assessment or treatment. Risk also features within the MCA where restraining a person to prevent harm to them is justified if it is proportionate to the likelihood of that person suffering harm and its seriousness (MCA S.5). On its face, the MCA approach excludes consideration of harm to others focusing instead on the person. The ADM elements of the MCA inform decision-making in a person's best interests (or, with ADRT or LPA, substitutes for it) and, in principle, an MCA ADM-D can enable a person to specify the harms which they regard as serious or significant - the personal harms.

### 6.3 Personal and public aspects of harm

We identify two ethical aspects of risk (or likelihood of harm) relevant to ADM which policy should reflect and provide cases to illustrate these points:

#### 6.3.1 A personal aspect: enabling individuals to identify what constitutes meaningful harm for them

i. One example is the service user who, based on past experience, considers coercion from mental health services to have been harmful. She has experience of her mental health deteriorating and harmful consequence from this. However, she has also experienced compulsory inpatient psychiatric admission and medication, and, on balance, feels this experience was traumatic and more harmful to her overall mental health. Her negative experience of admission means that she is often reluctant to seek help from services in the early stages of a crisis. She would like to use ADM-D to refuse medication which has significant side effects for her and admission to hospital. She believes this would relieve the fear of compulsory admission and mean she seeks help earlier and therefore experiences less deterioration in her mental health. The service user organisation ‘Hearing Voices Network’ conducted a survey and created an alternative review of the MHA (Hart & Waddingham, 2018) which reflects this perspective.
ii. A contrasting example is a service user such as P described by Gergel and Owen (2015). P is often only detained in hospital after he has become severely unwell and experienced significant harm from episodes of mania. In crisis he refuses admission but in retrospect wishes he had been detained earlier and so avoided harm to his property, relationships and disruption to his employment. P considers the harm of compulsory detention is outweighed by the harms of untreated mania in the community. He would like to use an ADM-D to request compulsory admission and treatment (self-binding ADM-D). Farrelly et al. (2014) remark on some cases of joint crisis plans being used to achieve this end.

#### 6.3.2 A public aspect: enabling the public body (community) to protect its interests

i. One scenario is third party harm occurring as a direct result of treatment refusal in an ADM-D (as discussed in Section 3.2.4). Clinicians often express concern about this possibility in psychosis though there are few cases of it to our knowledge. One American case (Fleming v. Reid (1990), 73 O.R. (2d) 169 (Dist. Ct.)) exemplifies some of these issues. Reid had a diagnosis of schizophrenia and substance abuse. He was detained after committing robbery and found not guilty by reason of insanity. His detention was continued due to concerns about risk of harm to himself and others. He was determined to be not competent to consent to psychiatric treatment and when members of his family withdrew from their roles as substitute decision makers the Official Guardian was appointed. An investigation uncovered that Reid had expressed a prior wish, when competent, to refuse antipsychotic medication. Therefore, the Official Guardian refused on Reid's behalf. His detention was prolonged and despite improvement when he was medicated (when the opinion of the Official Guardian was challenged) he continued to refuse antipsychotics. When the Official Guardian's opinion was upheld antipsychotic medication was withheld. After 9 months Reid's mental state deteriorated resulting in violent behaviour and multiple seclusions. Eventually, his brother stepped forward. It is unclear what happened next legally but antipsychotic medication was restarted, Reid improved dramatically on medication and was discharged and able to live independently in the community (Solomon et al., 2008).
ii. Another scenario is public cost. In the Starson case (detailed in Section 3.2.4) an individual was legally detained for long periods on the grounds of mental illness and risk of harm yet treatment was not possible due to his capacity to refuse. This case highlights the potential unintended consequence of ADM-D being be used to refuse treatment but not detention. Such an individual could be unnecessarily deprived of their liberty for prolonged periods at community cost.
iii. A final scenario is insurmountable ethical controversy within a community. Kerrie Wooltorton used an ADM-D to refuse potentially life-saving treatment following self-harm (recounted in Section 3.2.3). Her presentation created a profound dilemma for the clinical team she presented to and it continued in the public debate that followed.

## 7 Meeting the challenge and getting the balance

There is a cultural movement toward autonomy in healthcare with widespread agreement that ADM policy should be advanced. Precedent autonomy (one facet of autonomy) is a principle of self-determination that should apply to mental health decision making as to physical health decision making but in a way that recognises differences as well as similarities. There is evidence that mental health service users want ADM-Ds and some evidence, especially when there is clinical buy-in, that mental health ADM-D can achieve positive clinical outcomes such as improved working alliance and reduced use of coercion.

Public interests need to be considered and we have attempted to identify what they are and how they can be protected. Public protection anxiety however must not be the tail that wags the dog and the evidence on the clinical feasibility of most mental health ADM-Ds puts this anxiety into perspective.

It is important to recognise, however, that unlike the other jurisdictions we have surveyed, England and Wales is not a blank slate when it comes to statutory provision for mental health ADM-D. As well as some entirely informal clinical templates in use, the MCA has brought in 4 statutory concepts:

i. Written statements made with capacity
ii. ADRT
iii. LPA health and welfare
iv. Existence, validity and applicability

Within one of the MCA's main statutory concepts - best interests - there is also a requirement that a person's ‘past wishes and feelings’ on a matter be *considered.* This is the case regardless of a person's mental capacity at the time the wishes and feelings were expressed, although greater weight will be given to written statements made when the person had capacity. Informal ADMs (which will have been made without formal mental capacity assessment) therefore need consideration in best interests. How much weight they are given will depend on the context in which they were made and on other factors that need to be considered within best interests assessment.

The MHA does not have statutory concepts (excepting for ECT and CTOs) but does have a chapter in its code of practice on “wishes expressed in advance” which refers to mental capacity. In addition the code of practice contains a “empowerment and involvement” principle which states that patients should be fully involved in decisions about care, support and treatment (Department of Health, 2015, Chapter 9).

We now consider two basic questions in developing ADM policy in the MHA.

### 7.1 Formality or informality of ADM in MHA?

Should there be formal rules on ADM in the MHA, or should mental health ADM be mainly left to informal arrangements/soft law?

On the one hand it is arguably too legally complex (Berghmans & van der Zanden, 2012). The effect of trying to do it using statute will be to inhibit update of ADM-D and create administrative burden. Plus, service users express interest in informal arrangements (Hindley et al., 2019).

However, ADM is a human rights issue (Ward, 2017) so there should be codification of that right. ADM is already codified in MCA and having no codification in MHA (except for ECT and CTO) creates a stark and confusing interface. Most stakeholders want change (e.g. MHA alliance survey (Mental Health Alliance, 2017)). In addition, informal kinds of ADM have not delivered on their anticipated benefits (e.g. CRIMSON study (Thornicroft et al., 2013)).

Our recommendation is that there need to be some formal rules in MHA because:

i. There is evidence service users and their families, on balance, want it.
ii. The MHA already includes some rules, but they are confusing.
iii. New rules can be used to reduce some of the difficult MCA/MHA interface issues.
iv. Mental health ADM has not advanced to the level of expectation and some new rules (with their statutory power) could mobilise resources and help clinicians and patients feel more certain without undue administrative burden.

### 7.2 MCA/MHA “fusion” on ADM?

Given there should be some formality for mental health ADM, should separate provision be adopted or should capacity-based ADM apply, identically, across mental health and physical health such as in Northern Ireland's Mental Capacity Act (sometimes called “fusion”)?

Arguments for adopting ‘fusion’ law include, firstly, that the current policy position on ADM largely comes from the MCA. Therefore, given the policy is to be expanded to mental health, it makes policy sense to carry across what already exists. Secondly, ADM reflects autonomy rights and it is discriminatory not to have parity across MCA and MHA.

However, arguments against fusion law assert that there is only (to our knowledge) one jurisdiction which has carried across the MCA ADM-Ds (or similar) to traditional mental health laws. This is Northern Ireland. But there is no implementation experience in Northern Ireland of this policy. Therefore, it would be an untested policy position for the MHA to adopt all the MCA's ADM concepts. In addition, as our analysis has shown, there are differences between mental health ADM and physical health ADM. Some of the analysis suggests mental health ADM requires considerations (e.g. requests, self-binding, prospective evidence of the existence, validity and applicability of an ADM) which are underdeveloped in the MCA. Also, some of the analysis suggests mental health ADM does not have the purpose that it serves under the current MCA regime (e.g. ADRT life sustaining, attorneys becoming solely responsible for compulsory treatment decision-making) or that mental health ADM does not need to consider third party harms. Not every differentiation of treatment will constitute discrimination, if the criteria for such differentiation are reasonable and objective and if the aim is to achieve a purpose which is legitimate.

Our recommendation to the IRMHA – broadly accepted by the Review – was that the MHA can adapt some of the MCA's ADM concepts with some reasonable adjustments for the mental health context in which the MHA is applied. We also recommended that the MHA introduces some new positive rules on mental health ADM (not in the MCA) which are appropriate to the mental health context. Most of these have had some testing in Scotland and have been broadly well received.

We believe a balance can be struck which:

i. Enables a culture change on written statements made with capacity such that they are developed within clinical services and involve joint working on requests as well as potential refusals.
ii. Enables mental health ADRT within public interests.
iii. Advances the mental health attorney agenda using a “nominated person” system.
iv. Gives patients more insurance that well thought through mental health ADMs will be given *particular weight* by introducing an accountability system to authenticate ADMs made with decision-making capacity, without duress and which are applicable.

We follow on now with the specific recommendations we made for reform and then summarise the current position in England.

## 8 Conclusion: specific recommendations

### 8.1 Legal changes

Our specific recommendations for statutory change:

8.1.1 Carry across MCA *written statements made with capacity* into the MHA such that these statements should be *given particular weight* by clinical decision makers. Written statements could include requests as well as refusals and there would be a policy expectation that services users would write these in consultation with, or after discussion with, mental health professionals (unless good reasons otherwise).
8.1.2 Carry across MCA *ADRT* into MHA but with limitations inserted into the MHA. The limitations could be expressed as follows:
8.1.3 Treatment for mental disorder can be provided notwithstanding it conflicts with an existing, valid and applicable ADRT where:

i. The treatment is intended to secure the life of the individual;
ii. The need for treatment arises from, and is proportionate to, the risk that not providing that treatment would pose to third parties.

In both cases, notifying relevant people/a body what they have done and why. The sorts of reasons being:

a. ECHR Article 2 (life) and ECHR article 8 (autonomy) rights are in conflict and there is a clear case that article 2 rights are weighing more heavily – e.g. emergency lifesaving situations
b. The ECHR article 8 (autonomy) right is qualified by a clear public protection consideration – e.g. significant overt aggression as a consequence of ADRT or a criminal court order such as MHA Section 41 restrictions or hybrid orders.

For the sake of clarity, we add that were there disagreements about the existence, validity and applicability of an ADRT needing clarification by a court, the clinical decision-maker could treat for mental disorder according to the usual provisions of the MHA whilst waiting for the court clarification.

These limitations would replace the existing part IV MHA S.62 rules on “urgent treatment”. We think they are a better statement of the legitimate public interest and do not conflate public interest with urgency.

8.1.4 We considered the question of whether admission to hospital should qualify as “treatment” within the meaning of ADRT. In other words, we considered whether people should be able to make advance refusals of admission to hospital. We think advance decisions to refuse admission to hospital would: a) avoid ethically problematic “Starson” cases (Section 3.2.4) in which a legal duty not to treat on the basis of an ADRT is *combined* with a legal duty to not discharge (i.e. to maintain admission to hospital), b) encourage clinicians to communicate to patients what care and treatment purpose hospital admission may be serving (e.g. short term respite in a place of safety rather than medication), and c) give patients who hold strong negative views about admission a meaningful legal instrument, within public interests, to determine that admission to hospital be used, in their case, only as a last resort.
8.1.5 Avoid setting the statutory detention criteria at the threshold of ‘imminent risk of suicide or violence’. Doing this would seriously impede ADMs from informing the judgement about whether the harm criteria for compulsory admission/treatment are met. For example, an ADM drawn up with detailed personal knowledge of patterns of mental health deterioration with resultant harms (including significant harms to health and welfare) may legitimately seek to inform MHA assessment and caution against delays. ‘Imminent risk’ thresholds may force assessors to delay MHA assessments in the face such ADMs. Also, an ‘imminent’ risk threshold would give no meaningful role for an ADM that had the aim of advance refusal of hospital admission (Section 8.1.3). This is because the criteria for overriding advance refusal of admission would be identical to the ‘imminent’ risk criteria for detention - making such advance refusal redundant. As in Sections 6.3.1 and Section 8.1.3 above, we recommend that such ADMs based on personal knowledge of admissions being harmful or a wish for admission to be used only as a last resort should be permissible within reasonable public interests considerations.
8.1.6 Write a system of “nominated person” similar to Scotland's “named person” into the MHA rather than carry across the MCA LPA system. If there is an existing MCA LPA health and welfare appointed, then the LPA, with agreement, becomes the “nominated person”.
8.1.7 Write an ‘authentication’ system for ADM into MHA similar to Scotland's ‘certification’ system. Define a group needed to authenticate existence, validity and applicability for ADRTs, written statements and nominated persons (basically trained professionals similar to those defined in the Scottish legislation). Specify that authentication is not a requirement for a written statement, ADRT or nominated person but that authentication, if completed, brings in a new statutory duty for the ADM to be registered on a national database and for any decision maker overriding the ADM to notify relevant people/body (see Section 8.1.9) giving reasons why.
8.1.8 Recognise that informal ADMs (or statutory ADMs which are not authenticated) should be recognised as ‘past wishes and feelings’ and considered in MHA decision making as they are in MCA decision making.
8.1.9 Introduce a specialist, multidisciplinary body for England and Wales (similar to the Mental
  Welfare Commission for Scotland) to oversee ADM authentication such that it can respond to individual cases, produce guidelines for service users and clinicians and work with the inspectorate body of the CQC.

### 8.2 Code of practice changes

Our recommendation for the MHA code of practice:

#### 8.2.1 Guidelines for written statements made with capacity

These should be directed toward people who have broad trust in mental health services and want to work jointly with mental health services (including on self-binding). There should be an expectation that NHS health care teams engage in collaborative work to find areas of agreement and be willing to assess for authentication (without charge) as part of good clinical practice. Template proformas for written statements should be developed and evaluated with specialised templates for self-binding written statements. Administrative simplicity should be an aim and there should be clear communication that written statements are not legally binding in the same way that an ADRT is.

#### 8.2.2 Guidelines for ADRT

These should be directed to service users who want to be able to ensure that refusals of specific types of medication (e.g. those which have caused problematic side effects) or ECT would be legally binding within the public interest limits.

They should explain the difference between ADRT (legally binding) and written statements involving refusals (not legally binding) for those seeking to use both types of ADM with their mental health teams.

ADRT guidelines should also direct toward service users who have low levels of trust in compulsory treatment or mental health service values (e.g. on risk, protection imperative, medication, etc.) to clarify how ADRTs can be used to shape mental health treatment. Guidelines should be developed to communicate to patients what the public interests are (life and third party harm) to prevent misunderstandings and aid working alliance. Guidelines should also be developed to add clarity for clinicians on what their protections from liability are within a rare working alliance where a service user's ADRT extends to inpatient treatment and most or all medications (i.e. that such an ADRT's effect is to shift the criteria for compulsory treatment from the default MHA criteria to imminent and direct dangerousness criteria).

#### 8.2.3 Guidelines for nominated persons

These need to recognise attorney burden and the issues of safeguarding coercion/deprivation of liberty in a MHA context. There would also need to be rules for those who do not appoint a nominated person or for those without capacity to appoint such a person.

#### 8.2.4 Guidelines for authentication: existence, validity and applicability

The terms existence, validity and applicability are legal terms of art in the MCA and need to be clarified for non-specialists. Guidelines should make it clear that authentication should cover issues of fluctuating DMC, vulnerability and duress and how to ensure refusals/requests are adequately specified and contemporaneous. Guidelines should also make clear that informal ADMs or statutory ADMs which are not authenticated also have a role. They should be recognised as ‘past wishes and feelings’ and given individual contextual consideration as in the best interest test of the MCA.

### 8.3 Implementation

Our recommendations for implementation:

i. The MHA in its principles should reinforce the NHS duty to provide for mental health and include a reference to ADM.
ii. The MHA should empower a specialised body for England and Wales (similar to the Mental Welfare Commission Scotland) to facilitate awareness of mental health ADM-D, provide case review and guideline development.
iii. The IRMHA should advocate that professional bodies such as the Royal College of Psychiatrists and the British Psychological Society are involved in the passage of the changes and take a lead in professional training development.
iv. The IRMHA should advocate that the Department of Health provide Mental Health Trusts with up to date models of ADM-D implementation. An example is the 3 approaches model taken by the state of Virginia in the US which in 2015 was the first US state to commit to a full implementation of a mental health ADM policy (Zelle, Kemp, & Bonnie, 2015). The 3 approaches are: 1) 1:1 education and facilitation by licenced staff, 2) 1:1 education and facilitation by peers, 3) group education and facilitation.
v. The IRMHA should encourage leading mental health and service user led charities to participate in the development of ADM-D templates.

### 8.4 The current position in England

The IRMHA created a vital opportunity for mental health legislation in England and Wales to join other jurisdictions in embracing legal provision for mental health ADM. In line with our recommendations, the IRMHA's final report (Wessely et al., 2018, p86–87) recommended that service users should be able to nominate a person of their choice to be involved in their care when detained under the MHA and that statutory “advance choice documents” should be created that enable people to make a range of choices and statements about their inpatient care and treatment and that these should be piloted to identify the detail needed to inform/impact practice.^4^

The IRMHA report considered that treatment preferences, refusals, information on circumstances that may indicate loss of DMC and personalised information on harms were all relevant to advance choice documents. There were also recommendations for a system of DMC authentication (Section 8.1.7), national and local databases and a stipulation that authenticated advance choice documents must form part of the treatment plan (or if they are not the responsible clinician must state the reasons why this is not the case). It was also recommended that tribunals should reference the advance choice documents. The IRMHA did not embrace all ADM characteristics of the MCA. Nominated persons do not have the legally binding decision-making authority of LPAs for health and welfare and advance choice documents involving refusal do not have the legally binding nature of ADRTs. On refusals the IRMHA recommended that authenticated advance refusals of ECT can only be overturned by a judge and that authenticated advance refusals of other standard treatments could only be overturned with the agreement of a second opinion doctor or: the rather broadly phrased, criteria “immediately necessary to save life, to prevent a serious deterioration in condition, to alleviate suffering or to prevent violence or damage to self and others” are met (Wessely et al., 2018, p225). The IRMHA report did not recommend a mental welfare commission to oversee ADM-Ds and no specific relevant recommendations on code of practice changes or implementation were given.

The UK government followed publication of the IRMHA report with a statement, on the same day, that a Mental Health Bill will be brought forward to parliament. The Government committed, immediately, to accepting the Review's recommendations on nominated persons and advance choice documents (Department of Health and Social Care, 2018); other recommendations will be considered in due course. Policy ground has thus been made on mental health ADM in England. The priorities now are to scrutinise the consultation documents and/or any Mental Health Bill when produced and detail the ADM recommendations further for purposes of code of practice change, implementation projects and guidelines.

In this paper we have seen how several key issues (physical vs. mental illness, self-determination vs public interest, MCA vs. MHA) converge in ADM. In England and other jurisdictions ADM looks set to play an increasingly important role in health policy and practice debates.

## Competing interests

Alex Ruck Keene was the legal adviser to the Independent Review of the Mental Health Act.

## Funding

This study was funded by Wellcome – grant number 203376/Z/16/Z.

## Figures and Tables

**Table:** 

Advance Decision Making	ADM
Advance Decision Making Document	ADM-D
Advance Decision to Refuse Treatment	ADRT
Community Treatment Order	CTO
Decision Making Capacity	DMC
Electroconvulsive Therapy	ECT
Independent Review of the Mental Health Act	IRMHA
Lasting Power of Attorney	LPA
Mental Capacity Act 2005	MCA
Randomised Controlled Trial	RCT
The Mental Health Act 1983	MHA
United Nations Convention on the Rights of Persons with Disabilities	CRPD

## Appendix A Legal cases

1. Airedale NHS Trust v Bland (1993) AC 789
2. A local authority v E and others [2012] EWHC 1639 (COP)
3. An NHS Trust v Ms. X [2014] EWCOP 35
4. Fleming v. Reid (1990), 73 O.R. (2d) 169 (Dist. Ct.)
5. Nottinghamshire Healthcare MHS Trust vs RC [2014] EWCOP 1317
6. Re. C ((Adult Refusal of Treatment) [1994] 1 WLR 290)
7. Schloendorff v Society of New York Hospitals (1914) 211 NY 125
8. Starson v. Swayze, [2003] 1 S.C.R. 722

## Footnotes

1 Hereafter we refer to the English legal position, the position in Wales being governed by the Mental Health Act 1983 and certain Wales-specific provisions.

2 The relation between mental illness and physical illness also needs some clarifications:

o People with mental illness can face physical health decisions (e.g. a person with schizophrenia who faces a cancer decision, a person with a needle phobia who faces a caesarean section)
o Mental illness can have physical causes which overlap with physical illness (e.g. metabolic, infective, traumatic, etc.). So, a person with a physical illness can face connected mental health decisions (e.g. a person with Huntington's disease facing decisions about dementia; a person with diabetes facing decisions about hyperglycaemic or hypoglycaemic confusional states; or a person with a falling BMI facing decisions about worsening eating disorder).
o Physical illness can be caused by mental illness. So, people with a mental illness can face connected physical health decisions (e.g. a person with depression and a suicide attempt with paracetamol facing a decisions about liver transplantation, a person with severe dehydration resulting from delusions facing a decision about renal dialysis).

3 It is not claimed that the scenarios described are such that a clinician working in a physical healthcare setting would never encounter them. It is rather that these scenarios are routine occurrences in mental health care settings but rarer in physical healthcare settings and therefore demand explicit consideration.

4 The Mental Health and Justice project is conducting further research with potential to inform the implementation process by:

a. Consulting service users, their friends and family, lawyers, specialist social workers, consultant psychiatrists and care coordinators to create a pilot template for co-produced advance choices with a particular focus on enabling service users to employ ‘self-binding’ if they choose (Stephenson et al., 2019).
b. Implementing the template in clinical practice (planned for 2019/2020). The project will generate guidance which can feed into revision of the MHA code of practice and other guidelines.
